# Supplementary material for: Inflammatory and Humoral Immune Responses to Commercial Autogenous Salmonella Bacterin Vaccines in Light-Brown Leghorn Pullets: Primary and Secondary Vaccine Responses
Source: Vaccines (Basel). 2025 Mar 13;13(3):311. doi: 10.3390/vaccines13030311 (PMC11946197; doi:10.3390/vaccines13030311)
Supplement: Supplementary file 1 [file vaccines-13-00311-s001.zip › vaccines-3509836-supplementary.pdf]

Supplementary Table S1. Primer and probe sequences for target genes.

Target	Primer/ Probe	Sequence (5′-3′)[1]	Accession No.
28S[2]	Forward	GGCGAAGCCAGAGGAAACT	X59733
	Reverse	GACGACCGATTTGCACGTC	
	Probe	[FAM]-AGGACCGCTACGGACCTCCACCA-[TAM]	
IL-1β[2]	Forward	GCTCTACATGTCGTGTGTGATGAG	NM_204524.1
	Reverse	TGTCGATGTCCCGCATGA	
	Probe	[FAM]-CCACACTGCAGCTGGAGGAAGCC-[TAM]	
IFN-γ[3]	Forward	GTGAAGAAGGTGAAAGATATCATGGA	NM_205149.1
	Reverse	GCTTTGCGCTGGATTCTCA	
	Probe	[FAM]-TGGCCAAGCTCCCGATGAACGA-[TAM]	
IL-6[2]	Forward	GCTCGCCGGCTTCGA	NM_204628.1
	Reverse	GGTAGGTCTGAAAGGCGAACAG	
	Probe	[FAM]-AGGAGAAATGCCTGACGAAGCTCTCCA-[TAM]	
IL-12α[4]	Forward	TGGCCAAGGGACTCAACTG	NM_213588.1
	Reverse	ACCTCTTCAAGGGTGCACTCA	
	Probe	[FAM]-CCGCTGCAAACGAGGCACTCCT-[TAM]	
IL-4[5]	Forward	AACATGCGTCAGCTCCTGAAT	NM_001007079.1
	Reverse	TCTGCTAGGAACTTCTCCATTGAA	
	Probe	[FAM]-AGCAGCACCTCCCTCAAGGCACC-[TAM]	
IL-8[4]	Forward	GCCCTCCTCCTGGTTTCA	NM_205498.1
	Reverse	TGGCACCGCAGCTCATT	
	Probe	[FAM]-TCTTTACCAGCGTCCTACCTTGCGACA-[TAM]	
IL-10[4]	Forward	CATGCTGCTGGGCCTGAA	AJ621614
	Reverse	CGTCTCCTTGATCTGCTTGATG	
	Probe	[FAM]-CGACGATGCGGCGCTGTCA-[TAM]	
TGF- β1[6]	Forward	GGTTATATGGCCAACTTCTGCAT	NM_001318456.1
	Reverse	CCCCGGGTTGTGTTGGT	
	Probe	[FAM]-AGCGCCGACACGCAGTACACCA-[TAM]	
IL-17A	Forward	ACATGAACCAGGATACCAAAGTGA	NM_204460.1
	Reverse	TGGTCCTCATCGATCCTGTAATC	
	Probe	[FAM]-CCTTGATATCAGCAAACGCTCACTGGC-[TAM]	
IL-13[5]	Forward	CACCCAGGGCATCCAGAA	AJ621735
	Reverse	TCCGATCCTTGAAAGCCACTT	
	Probe	[FAM]-CATTGCAAGGGACCTGCACTCCTCTG-[TAM]	
TNF-α[7]	Forward	CGCTCAGAACGACGTCAA	MF000729.1
	Reverse	CCTTCTCAGCACCACGCCGTTA	
	Probe	[FAM]-CCGGCTCGTTGGTGTGGGA-[TAM]	

[1]Primers and probes were designed in-house using NCBI's Primer-BLAST tool unless otherwise indicated; oligos were synthesized by Eurofins MWG Operon LLC, Huntsville, AL; [2]Sequences from Kogut et al. [74]; [3]Sequences from He et al. [75]; [4]Sequences from Smith et al. [76]; [5]Sequences from Avery et al. [77]; [6]Sequences from Falcon [78]; [7]Sequences from Rohde et al. [79]
